# Impact of Comorbidity Scores on the Overall Survival of Patients With Advanced Non-small Cell Lung Cancer: A Real-World Experience From Eastern India

**DOI:** 10.7759/cureus.30589

**Published:** 2022-10-22

**Authors:** Srikanth Goud M, Prasanta R Mohapatra, Sourin Bhuniya, Saroj Kumar Das Majumdar, Pritinanda Mishra, Manoj K Panigrahi, Shakti K Bal, Ananda Datta, Palanisamy Venkatachalam, Debopam Chatterjee

**Affiliations:** 1 Pulmonary Medicine and Critical Care, All India Institute of Medical Sciences, Bhubaneswar, Bhubaneswar, IND; 2 Radiation Oncology, All India Institute of Medical Sciences, Bhubaneswar, Bhubaneswar, IND; 3 Pathology and Laboratory Medicine, All India Institute of Medical Sciences, Bhubaneswar, Bhubaneswar, IND

**Keywords:** real-world study, eastern india, overall survival, comorbidities, advanced lung cancer

## Abstract

Introduction

Lung cancer is the most common cancer, and it is the leading cause of cancer-related death. Smoking is the most common risk factor for the development of lung cancer. There is a lack of data on the comorbidities and outcomes of advanced non-small cell lung cancer (NSCLC) in the eastern part of India. This prospective study evaluated the impact of comorbidity scores on overall survival (OS) in these patients.

Method

This prospective cohort study was conducted on newly diagnosed advanced NSCLC patients between June 2020 and April 2021. These patients were given platinum-based doublet chemotherapy guided by histology and targeted therapy based on molecular studies. Comorbidities were assessed using the Charlson Comorbidity Index (CCI), Simplified Comorbidity Score (SCS), and Adult Comorbidity Evaluation-27 (ACE-27). The outcome assessed was OS. Overall survival was calculated in days from the date of start of anticancer therapy to the date of last follow-up or date of death. All enrolled patients were followed at regular intervals whenever they visited the hospital and telephonically until April 2022. The patients who were alive on April 30, 2022, were censored. The survival probability and median OS were calculated by Kaplan-Meier analysis, and group differences in comorbidity scores were analyzed with the log-rank test. A Cox proportional hazard analysis was performed to look for factors affecting overall survival.

Results

A total of 114 patients were enrolled in the study period, and the mean age of patients was 56.54 ± 11.03 years. Most of the patients were males (68.4%), and 52.6% were smokers. Adenocarcinoma was the most common histology (73.7%), followed by squamous cell carcinoma (25.4%). The median OS was 127 days (95% CI, 60-193 days). 33.4% of the patients had a CCI score of 0, a CCI score of 1 was seen in 57%, and ≥2 scores in 9.6%. SCS scores ≤9 and >9 were seen in 92.1% and 7.9% of patients, respectively. The ACE-27 score was none in 41 subjects, mild in 59, moderate in 12, and 2 NSCLC subjects had severe ACE-27 scores. The median OS for patients with a CCI score of 0 was 275 days (95% CI, 7-543 days), 114 days (95% CI, 85-142 days) for subjects with a CCI score of 1, and 402 days (95% CI, 0-844 days) for patients with a CCI score ≥2 (log-rank p = 0.215). Individuals with an SCS score ≤9 had a median OS of 175 days (95% CI, 91-258 days), and the median OS was 92 days (95% CI, 80-103 days) for patients with an SCS score >9 (log-rank p = 0.302). Median OS of the patients with ACE-27 score 0,1,2,3 were 297 days (95% CI, 76- 517 days), 117 days (95% CI, 81-152 days), 87 days (95% CI, 49-124 days) and 66 days, respectively (log-rank p=0.457). There was no statistical significance between comorbidity scores and OS. Worse OS was independently associated with poor performance status Eastern Cooperative Oncology Group (ECOG) ≥2 (hazard ratio [HR] 3.266; 95% CI 1.785-5.978; p = 0.00), neutrophil-to-lymphocyte ratio (NLR) <3 (HR, 2.35 95% CI 1.18-4.702; p = 0.015) and patients who were given compassionate tyrosine kinase inhibitors (TKIs) (HR, 7.396 95% CI 3.531-15.490; p = 0.000).

Conclusions

In our study, the advanced NSCLC patients who were given chemotherapy or oral TKIs showed no significant influence of comorbidities on overall survival. Factors independently associated with the worst survival were poor performance status (ECOG ≥ 2), NLR < 3, and patients who were given TKIs on a compassionate basis.

## Introduction

Lung cancer cases are increasing worldwide, and management of advanced lung malignancy has various challenges in developing countries. Globally, there are about 2.20 million new cases, among 11.4% of total thoracic malignancy cases and 1.79 million deaths (18 % of total cancer deaths) as per 2020 GLOBOCAN [1]. Comorbidities in lung cancer patients may influence diagnosis and complicate treatment. The Charlson Comorbidity Index (CCI) is the most commonly used scoring system for assessing comorbidities in chronic diseases [2]. Simplified Comorbidity Score (SCS) is another validated score specifically for non-small cell lung cancer (NSCLC) [3]. There is conflicting information on comorbid conditions and lung cancer outcomes. Lung cancer treatment has several challenges regarding diagnosis, obtaining samples by various modalities, and cost issues. Comorbidities also alter the desired treatment for the patient's problems [4]. Second, the association between comorbidities and survival was evaluated. The impact of comorbidities on systemic anticancer therapy in advanced lung cancer patients in India is still poorly considered and requires improving our knowledge in this area. In our study, we aim to assess comorbidity scores and overall survival (OS) in advanced non-small cell lung cancer patients and find an association between comorbidity scores and survival in these patients. This abstract was presented as an e-poster at the IASLC World Conference on Lung Cancer on August 6-9, 2022, in Vienna, Austria.

## Materials and methods

This prospective cohort study was conducted in the Department of Pulmonary Medicine and Critical Care, AIIMS, Bhubaneswar. The patients were enrolled between July 01, 2020, and April 30, 2021. All patients were followed up until April 30, 2022. The study participants included in the study were proven advanced NSCLC patients. Patients older than 18 years with lung cancer, confirmed by cytology or histopathology; patients with NSCLC with advanced disease (stages III B to IV); and patients willing to provide informed consent were included in the study. Exclusion criteria were diagnosed NSCLC patients with stage I to IIIA, malignancies other than NSCLC, like small cell lung cancer patients, and other neuroendocrine tumor patients. The approval of the study was obtained from the Institutional Ethics Committee of the All India Institute of Medical Sciences, Bhubaneswar (IEC/AIIMS BBSR/PG Thesis/2020-21/34).

Our study used three distinct scoring methods to record the participants' comorbidities. Comorbidities were measured by the CCI, SCS, and Adult Comorbidity Evaluation 27 (ACE-27) in the present study. These scores were recorded before the start of chemotherapy/tyrosine kinase inhibitors. Various validated scales assess comorbidities; some may not be specific to cancer patients. The Charlson Comorbidity Index is the most straightforward comorbidity assessment tool, validated in 1987 by Charlson et al. [2]. It was created to discover factors that increased the probability of one-year mortality in a cohort of hospitalized medical patients. It has 19 descriptors with a maximum score of 35. A simplified comorbidity score is a comorbidity index specifically studied in lung cancer. It has seven different descriptors, and the maximum score is 20. The predictive value of SCS remains unknown, with few published studies and inconsistent results [5]. A survey by Jacot et al. [6] in NSCLC showed increased mortality with high SCS (score >9). In contrast, there was no association between mortality and SCS in studies conducted by Girones et al. [7] and Ball et al. [8]. In our study [9], ACE-27 was also used for comorbidity scoring [9]. It contains 27 comorbid parameters with various grades. Permission has been taken from the developers of CCI, SCS, and ACE-27 to use them in this study.

Details of baseline investigations and data from imaging studies as part of the staging and evaluation of lung cancer were recorded by patient interviews and medical records with structured proforma. The lung cancer staging was done per the 8th edition of the TNM (tumor node and metastasis) classification [10]. Advanced lung cancer inflammation index (ALI) was evaluated by Jafri et al., and it was measured as body mass index (BMI) × albumin/NLR [11]. ALI was entered into enrolled patients before the start of anticancer therapy.

The chemotherapy regimens were platinum doublet therapy and radiotherapy was given to the patient per standard treatment protocol. The tumor response was studied by CT scanning and RECIST (Response Evaluation Criteria in Solid Tumors) criteria 1.1 [12]. CT chest was performed after four cycles of chemotherapy or when clinically indicated. RECIST was done after three months of treatment for patients who are on targeted therapy.

OS was calculated from the date of the start of either chemotherapy or oral tyrosine kinase inhibitors to death from any cause. All the enrolled patients who had started anticancer treatment were followed up for one year. In addition, they were followed up every time they visited for a chemotherapy cycle or outpatient department and every two months over the phone. All enrolled patients were followed regularly whenever they visited the hospital and telephonically until April 2022. The patients who were alive on April 30, 2022, were censored. An association of comorbidity scores and overall survival was performed in the study.

Statistical analysis

Data were entered in a Microsoft Excel sheet (Microsoft® Corp., Redmond, WA) and analyzed in SPSS statistical software 20.0 (SPSS Inc., Chicago, IL). Descriptive data were recorded as mean ± standard deviation (SD), median, and percentages. For statistical analysis, patients in the study group were divided into two groups: those with comorbidities and those without comorbidities. Comorbidity scores were also categorized into two or three groups. Comparison between groups was made by Fisher's exact test for categorical variables and an unpaired student t-test for continuous variables in two groups. Median OS was calculated using the Kaplan-Meier test. Patients were censored. They were known to be alive at the end of the study period. A Cox proportional hazard analysis was performed to look for factors affecting overall survival.

## Results

During the study period, a total of 114 patients were included. Table 1 shows the baseline characteristics and demographic features of the patients in the study. The mean age of patients was 56.54 ± 11.03 years. Most patients were males (68.84%), and females accounted for 31.6%. A total of 52.6% of the patients were smokers, and the remaining 47.4% were nonsmokers. The most frequent histology was adenocarcinoma (73.7%), followed by squamous cell carcinoma (25.4%), and one patient was diagnosed with the NSCLC-NOS type. About half of the patients have an ECOG score of <2, and ≥2 ECOG score was seen in 50.9% of the patients. Most patients were diagnosed by endobronchial biopsy (39.5%), followed by image-guided biopsy (27.2%), and 21.9% of the patient's diagnosis was established by pleural fluid cytology and cell block. Ultrasound-guided closed pleural biopsy and thoracoscopic pleural biopsy were utilized in establishing lung cancer diagnosis in 7.9% and 1.8% of the patients, respectively.

**Table 1:** Characteristics of patients included in the study IQR: interquartile range; ECOG: Eastern Cooperative Oncology Group; USG: ultrasonogram; EBUS TBNA: endobronchial ultrasound transbronchial needle aspiration; NSCLC: non-small cell lung cancer; NOS: not otherwise specified; EGFR: epidermal growth factor receptor; TKI: tyrosine kinase inhibitor; SCS: Simplified Comorbidity Score, CCI: Charlson Comorbidity Index, ACE-27: Adult Comorbidity Evaluation 27

Patients/s characteristics in detail	N (number of patients)	Percentage
Sex		
Male	78	
Female	36	
Median age (IQR)	57.5(50-65)	
Smoking status		
Never smokers	54	47.4
Smokers	60	52.6
Smoking index		
<250	14	23.3
>250	46	76.7
Never smokers	54	47,4
Smokers	60	55
Body mass index (kg/m^2^)		
<18.5	7	6.1
18.5-24.9	101	88.6
25-29.9	4	3.5
≥30	2	1.8
ECOG		
0	20	17.5
1	39	34.2
2	26	22.8
3	14	12.3
4	15	13.2
Mode of diagnosis		
Pleural fluid cytology and cell block	25	21.9
USG-guided closed pleural biopsy	9	7.9
Thoracoscopic guided biopsy	2	1.8
Endobronchial biopsy	45	39.5
EBUS TBNA	2	1.8
CT/USG guided biopsy	31	27.2
Histopathology		
Adenocarcinoma	84	73.7
Squamous cell carcinoma	29	25.4
NSCLC-NOS	1	0.9
CCI		
CCI 0	38	33.3
CCI 1	65	57
CCI >2	11	9.6
SCS		
≤9	105	92.1
>9	9	7.9
ACE-27		
None	41	36
Mild	59	51.8
Moderate	12	10.5
Severe	2	1.8
Treatment given		
First-line chemotherapy	66	57.9
TKIs	18	15.8
Compassionate TKIs	30	26.3
EGFR mutational studies		
EGFR positive	22	19.3
EGFR Negative	51	44.7
Not available/not done	41	36

Most advanced lung cancer patients had a BMI of 18.5-24.9, accounting for 88.6% of the patients (101/114), while a BMI of 18.5 was seen in 7% of the patients. The advanced lung inflammation index (ALI) was <18 in 58.8% of the patients, and ALI was >18 in 41.2 %. A neutrophil-to-lymphocyte ratio (NLR) at the start of treatment was noted. NLR <3 36% of the study participants were seen, and 64% of the patients had NLR ≥3. EGFR mutations were positive in 22 patients (19.3%), negative in 51 patients, and not known in 36% of the patients in our study. 57.9% of the enrolled patients were given first-line chemotherapy; tyrosine kinase inhibitors were given to 15.8%, and 26.3% were given compassionate TKIs.

In our study, 33.4% of the patients showed CCI scores of 0; CCI scores of 1 and ≥2 were seen in 57% and 9.6% of the patients, respectively. In the current study, SCS ≤ 9 was seen in 92.1% of the patients, and 7.9% had an SCS score of >9. ACE-27 was none in 41 patients (36%), 59 patients had a score of mild (51.8), and ACE-27 was moderate and severe in 12(10.5%) and 2(1.8%) patients, respectively.

**Figure 1:**
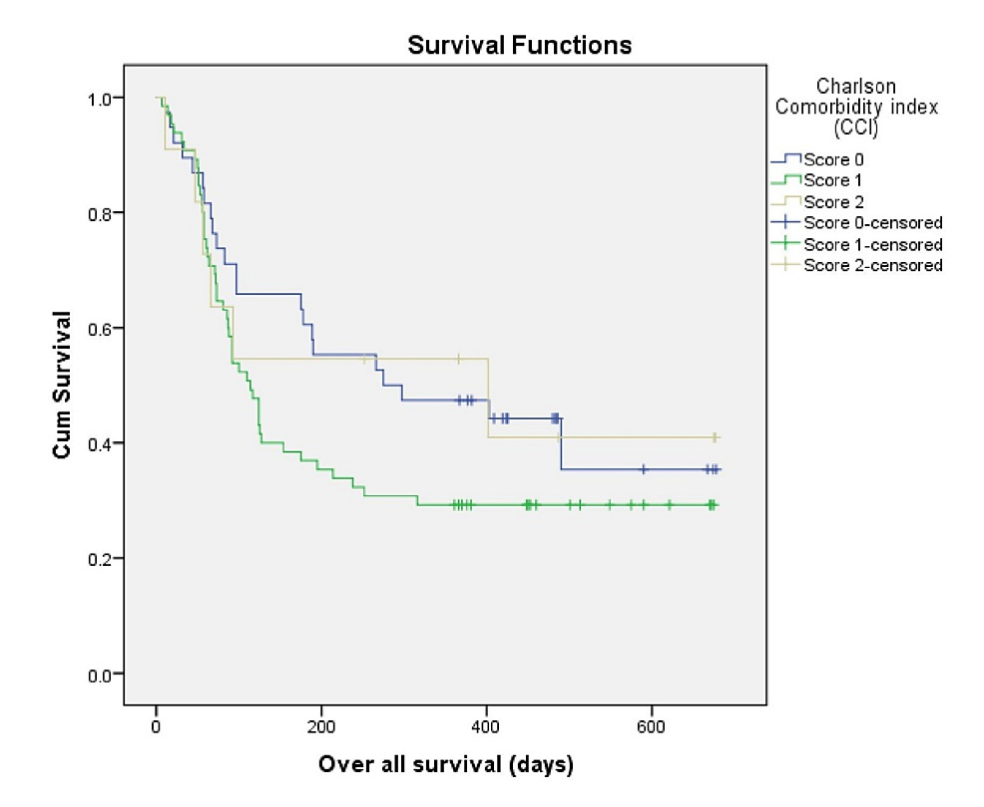
Kaplan Meier survival estimate according to Charlson Comorbidity Index

The median OS in our study was 127 days (95% CI, 60-193). There is conflicting evidence about the impact of comorbidities on clinical outcomes in advanced lung cancer. Therefore, our study has studied the association between comorbidities and OS. The median OS (Figure 1) for patients with a CCI score of 0 was 275 days (95% CI, 7-543 days), 114 days (95% CI, 85-142 days) for subjects with a CCI score of 1, and the median OS for patients with a CCI score ≥2 was 402 days (95% CI, 0-844 days) (log-rank, p = 0.215).

**Figure 2:**
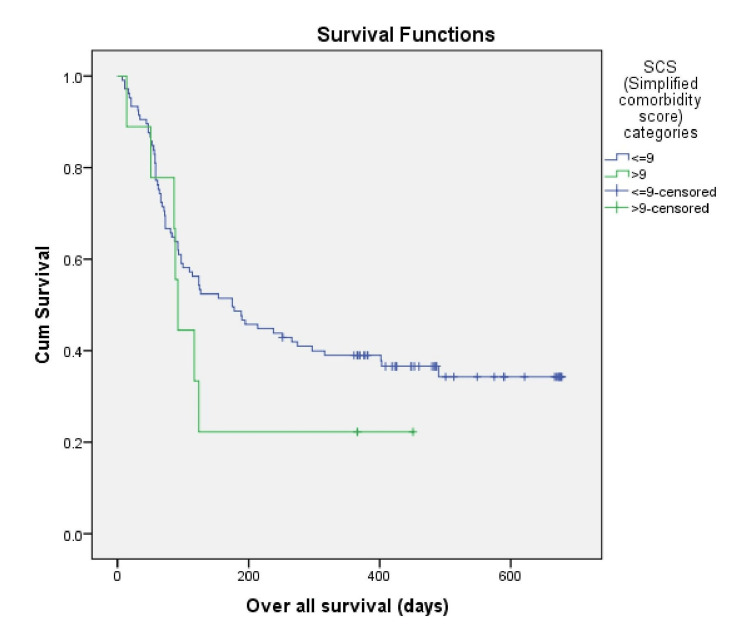
Kaplan Meier survival estimate according to SCS SCS: Simplified Comorbidity Score

**Figure 3:**
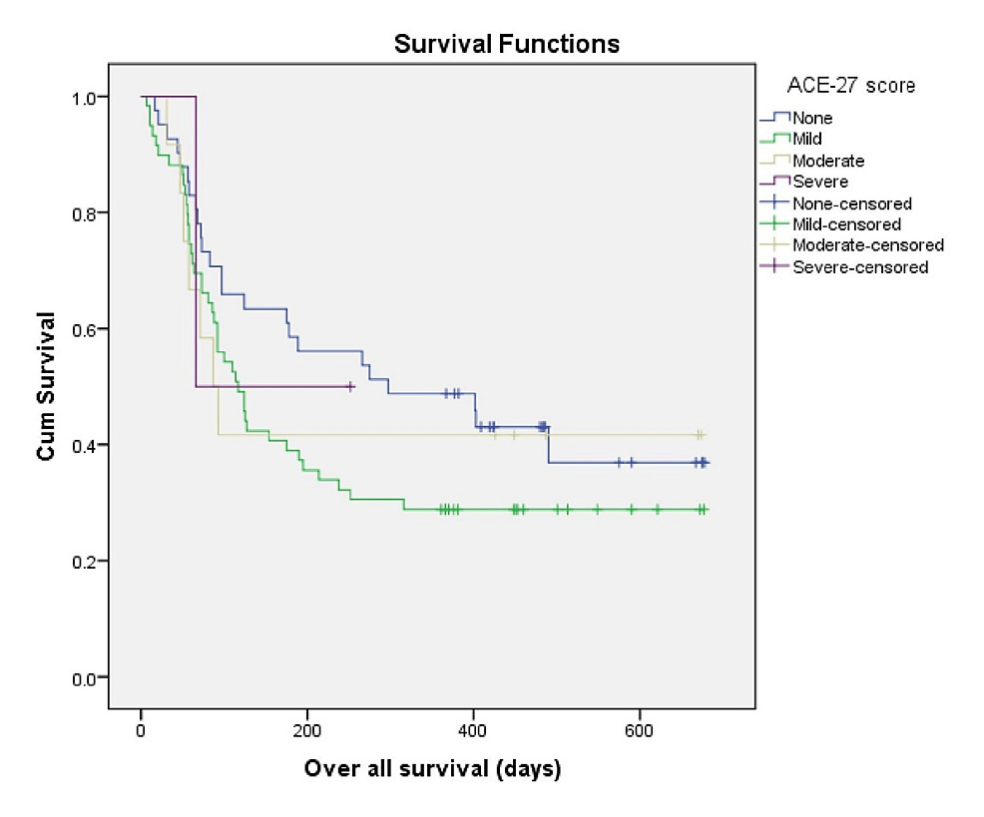
Kaplan Meier survival estimate according to ACE-27 ACE-27: Adult Comorbidity Evaluation-27

Individuals with an SCS score ≤ 9 had a median OS of 175 days (95% CI, 91-258 days), and the median OS was 92 days (95% CI, 80-103 days) for patients with an SCS score of >9 (log-rank p = 0.302) (Figure 2). The median OS of the patients in our study with an ACE-27 score of 0,1,2,3 were 297 days (95% CI, 46-547 days), 117 days (95% CI, 81-152 days), 87 days (95% CI, 49-124 days) and 66 days, respectively (log-rank, p=0.457). There was no significant difference between comorbidity scores and OS in these patients (Figure 3).

A Cox proportional hazards regression analysis was performed to assess the factors associated with worse overall survival (Table 2). In our study, poor performance status (ECOG≥2), NLR<3, and patients who were given compassionate TKIs had worse survival. Comorbidity scores (CCI, SCS, and ACE-27), patient age, and histology type did not influence OS after multivariate analysis.

**Table 2:** Factors affecting overall survival using unadjusted Cox proportional hazards regression analysis. HR: hazard ratio; CI: confidence interval; CCI: Charlson Comorbidity Index; SCS: Simplified Comorbidity Score; ACE-27: Adult Comorbidity Evaluation-27; ECOG: Eastern Cooperative Oncology Group; NLR: neutrophil-lymphocyte ratio; ALI: advance lung cancer inflammation index; NSCLC: non-small cell lung cancer; NOS: not otherwise specified; ECOG: Eastern Cooperative Oncology Group; TKI: tyrosine kinase inhibitor.

Variable	HR (95% CI)	P-value
CCI		
0	1	
1	0.767 (0.265–2.218)	0.624
≥2	1.861 (0.525–6.593)	0.336
SCS		
<9	1	
≥9	0.948 (0.415–2.165)	0.898
ACE-27		
None	1	
Mild	0.912 (0.339–2.453)	0.856
Moderate	1.449 (0.471–4.453)	0.518
Severe	0.214 (0.02–2.335)	0.206
Age		
<60	1	
>60	1.238 (0.731–2.096)	0.710
ECOG		
<2	1	
≥2	3.266 (1.785–5.978)	0.000
NLR		
<3	1	
≥3	2.355 (1.180–4.702)	0.015
ALI		
<18	1	
>18	0.404 (0.202–0.809)	0.011
Smoking status		
Yes	1	
No	0.710 (0.389–1.293)	0.710
Histopathology		
Adenocarcinoma	1	
Squamous cell carcinoma	0.688 (0.368–1.288)	0.242
NSCLC-NOS	0.00 (0.00–1.443)	0.970
Treatment given		
First-line chemotherapy	1	
TKI	0.922 (0.395–2.154)	0.922
Compassionate TKIs	7.396 (3.53–15.49)	0.000

## Discussion

In this prospective cohort study, we found comorbidities were frequent in patients with advanced NSCLC. Adenocarcinoma is the most frequent histology, followed by squamous cell carcinoma. OS was 127 days, which is low compared to other studies in India. The lower OS in our study can be due to the inclusion of advanced NSCLC with all performance scores, and more than half of the patients had ECOG scores>2. We found no significant impact on comorbidities or OS among these patients. In our study, poor performance status (ECOG ≥ 2), NLR<3, and patients who were given compassionate TKIs had worse survival.

In the present study, 33.4% of the patients had a CCI score of 0, a CCI score of 1 in 57%, and a CCI score of ≥2 was seen in 9.6%. In a study conducted by Singh et al. [13] that included both NSCLC and SCLC patients, the CCI score was 0 in 37% of the patients, 40.8% of the patients had a CCI score of 1, and the rest of the patients had a CCI score of ≥2.

In our study, the SCS score was ≤9 in 92.1% of the patients, and 7.9% had an SCS score of > 9. In their study, Singh et al. [13] showed that the SCS score was ≤9 in 72.7% of the patients, and the rest had SCS scores >9. This study included both SCLC and NSCLC and all stages of the disease. The results of our study are inconsistent with the above research as we had only advanced NSCLC patients.

ACE-27 was none in 36%, mild in 51.8%, and ACE-27 was moderate and severe in 10.5% and 1.8% of the patients, respectively. In a study done by Piccirillo et al. [9], the comorbidity level was none in 31.2%, 29.5% had a mild level of comorbidity, 25.4% showed a moderate score, and 14.2% of patients had a severe ACE-27 score. The results of our study were similar only in finding the absence of comorbidities in NSCLC. The severity of comorbidity in our study was not identical to the above research. It could be due to different smoking patterns and the high prevalence of non-communicable diseases in western countries compared to India.

The median OS in our study was 127 days. In a survey by Amar et al. [14], the median OS was six months in advanced NSCLC patients. The median OS was 7.6 months in a study by Murali et al. [15]. In a study in south India in a tertiary care center by Bala et al. [16], the median OS was 12.1 months. In a study by Garg et al. [17], the median OS was 11.7 months. In our study, the median OS was less than in other Indian studies, possibly due to the inclusion of patients with ECOG scores of 0-4 and possible delayed presentation and treatment initiation due to the COVID-19 pandemic and lockdown period. The COVID-19 pandemic significantly impacted cancer care, as shown in the research by Joode et al. [18]. Studies have shown that reducing patient delays in diagnosis and treatment is associated with improved survival in NSCLC [19]. The recruited patients were followed up for a shorter duration of time, and it might have influenced the overall survival. The results of our study may not be generalized due to the small sample size, single-center analysis, and enrollment of patients with poor performance status. The present study's findings could reflect real-life challenges of oncology care during the COVID-19 pandemic.

There is conflicting evidence about the impact of comorbidities on clinical outcomes in advanced lung cancer. A study by Singh et al. [13] on lung cancer that included both SCLC and NSCLC concluded that comorbidities do not influence lung cancer clinical outcomes. Zhao et al. [20] showed that overall survival with CCI <9 was 20.84 months, and ≥9 CCI score had OS of 17.80 months. The CCI score was a significant predictive factor for OS in advanced NSCLC patients with negative EGFR and ALK mutations in this study.

Individuals with an SCS score ≤9 had a median OS of 175 days and the median OS was 92 days for patients with an SCS score >9. A study conducted in North India by Singh et al. [13] showed the presence of comorbidities (SCS) was not associated with clinical outcomes in lung cancer patients. In a study by Alexander et al. [5], the median OS in stage IIIB was 13 months and 10 months for stage IV disease. This study showed in a cohort of NSCLC patients that SCS was not a significant factor in overall survival. Our study showed no significant difference between comorbidity scores using ACE-27 and OS.

A Cox proportional hazards regression analysis was performed to assess the factors associated with worse overall survival. In our study, poor performance status (ECOG≥2), NLR<3, and patients who were given compassionate TKIs had worse survival. Comorbidity scores (CCI, SCS, and ACE-27), patient age, and histology type did not influence OS after multivariate analysis. A study was performed by Zhao et al. [20] to look at the association between CCI and survival in advanced NSCLC. Male gender, smoking, and CCI score (<9 and >9) were significantly associated with outcomes. This study concluded that in advanced NSCLC patients, the CCI score is an independent prognostic factor of OS. A retrospective study was conducted by Joobeur et al. [21] on young NSCLC patients. They showed that performance status ≥2 and high CRP concentrations are associated with poor prognosis. Prognostic factors were assessed by Su et al. [22] in older patients who were diagnosed with advanced NSCLC, and they concluded that performance status and disease stage were independent factors. The study performed at the Adyar Institute Chennai, India, by Murali et al. [15] showed that factors associated with poor OS were non-adenocarcinoma histology, performance status >2, and stage of the disease. In our study, patients with ALI >18 had better OS than those with ALI <18. The results of our study also showed that poor performance status was associated with poor survival, which is consistent with the above studies.

We have encountered difficulties and issues in the study, mainly due to COVID-19 and the lockdown period. There has been a substantial delay in the necessary investigation and diagnostic and interventional procedures. Imaging and other metastatic workups were also delayed in some subjects. Patients got infected with COVID-19 before and during chemotherapy. During the lockdown, the patients were being monitored through telemedicine consultation. The scheduled follow-ups were delayed due to COVID-19 testing before chemotherapy, the patient's hesitancy to visit the hospital, and the presence of COVID-19 infection in the patient or any other family member.

It is the only study in Eastern India in which comorbidities were assessed prospectively so that comorbidity profiles would not be missed, as most of the previous literature is on retrospective charts/records. Low overall survival in our study may reflect the need to improve cancer care services even when pandemics are prevalent. Our study also had some limitations. Due to the severe COVID pandemic, we could not recruit more patients, leading to smaller sample size. An extended lockdown period due to pandemics might contribute to delayed referrals, presentation, and diagnosis, which may influence overall survival. Due to the redistribution of the workforce in the hospital, particularly among pulmonologists during COVID, there was a delay in diagnostic and staging procedures in the hospital. The delay in each step led to the progression of diseases and poor performance status. Finally, molecular analysis of the few patients was unavailable as our institute does not have the facility to perform mutational analysis.

## Conclusions

In this prospective cohort study, comorbidities are frequent in advanced NSCLC patients as assessed by different scales. There is no significant impact on the presence of comorbidities and overall survival among these patients. The low overall survival in our study could be the effect of COVID-19 on cancer care. Optimizing the management of comorbidities and cancer-specific treatments for NSCLC may improve outcomes. Our study showed that poor performance status is associated with worse survival irrespective of comorbidities, but studies with a larger sample size are required to assess factors associated with overall survival.
